# Characterization of a novel galectin in *Sarcoptes scabiei* and its role in regulating macrophage functions

**DOI:** 10.3389/fmicb.2023.1251475

**Published:** 2023-08-24

**Authors:** Ran He, Qian Zhang, Luyang Xu, Maochuan Guo, Xiaobin Gu, Yue Xie, Jing Xu, Zhaoli Shen

**Affiliations:** ^1^Department of Parasitology, College of Veterinary Medicine, Sichuan Agricultural University, Chengdu, China; ^2^College of Science, Sichuan Agricultural University, Ya'an, China

**Keywords:** *Sarcoptes scabiei*, galectins, macrophage, inflammation, mouse AD model

## Abstract

*Sarcoptes scabiei* (*S. scabiei*) endangers human and other mammalian health. There has been limited research into *S. scabiei* pathogenic mechanisms and the immunological interaction between *S. scabiei* and hosts. Galectins have critical roles in biological processes such as cell adhesion, signal transduction, and immune response mediation. Galectins of *S. scabiei* (*Ss*Galectins) were cloned, expressed, and identified, and their transcriptional levels in *S. scabiei* were measured at various developmental stages. Fluorescent tissue localization was performed on *Ss*Galectins of *S. scabiei* and scabies skin. A mouse AD model was constructed to evaluate the effect of r*Ss*Galectins on skin pathogenic changes. Quantitative polymerase chain reaction and enzyme-linked immunoassay were used to identify macrophage polarization-related components and investigate the immunoregulatory effect of r*Ss*Galectins on mouse macrophages. The results demonstrated that the *S. scabiei* infection causes macrophage infiltration in the scabies skin. The r*Ss*Galectins displayed strong reactogenicity, and distinct genes of the *Ss*Galectins were differently expressed in different developmental stages of *S. scabiei*. Fluorescence tissue localization revealed that the *Ss*Galectins were mainly in the mouthparts, intestines, and body surface. Additionally, *S. scabiei* could secrete *Ss*Galectins into the infected skin, proving that *Ss*Galectins were excretion and secretion proteins of *S. scabiei*. In the mouse atopic dermatitis model, cutaneous macrophage infiltration and inflammation increase after r*Ss*Galectins injection. Simultaneously, when r*Ss*Galectins acted on bone marrow-derived macrophages, M1 macrophage-related polarization factors IL-1β, IL-6, and inducible nitric oxide synthase all increased, demonstrating that r*Ss*Galectins can induce M1 polarization and produce pro-inflammatory cytokines. In conclusion, the *Ss*Galectins are involved in the pathogenic process of *S. scabiei* by regulating the polarization of host macrophages to the M1 type when *S. scabiei* invade the host and promoting the incidence and development of the host's inflammatory response. This study offers fresh light on the pathogenic process of scabies mites, investigates the immunological interaction mechanism between *S. scabiei* and the host, and offers new insights into *S. scabiei* prevention and therapy.

## Background

In 2021, the World Health Organization included scabies in its report on “Ending the neglect to attain the Sustainable Development Goals: a road map for neglected tropical diseases 2021–2030” (World Health Organization, 2022). Scabies is an infectious disease caused by *Sarcoptes scabiei* parasitizing the epidermis of humans and other mammals. *S. scabiei* mites are transmitted by contact and can infect more than 100 host species (El-Moamly, 2021). Scabies is mainly caused by host infection with scabies mites, which can lead to severe itching, scabbing, hair loss, and skin thickening and is generally followed by a bacterial skin infection, namely impetigo (Taiaroa et al., 2021). Improper handling can easily lead to severe complications, such as sepsis, kidney disease, rheumatic heart disease, and even death. Approximately 300 million people worldwide are affected by scabies every year (Reynolds et al., 2014). Similar to other neglected tropical diseases, the infection and morbidity of the disease are more pronounced in developing countries, especially in poor areas (Romani et al., 2015). *S. scabiei* mite infestation has a significant negative impact on the performance of economic animals in terms of output and quality of livestock products, in addition to negatively affecting their growth and development, causing severe global public health problems and substantial financial losses (Fischer and Walton, 2014; Bernigaud et al., 2019). At present, there is no vaccine against scabies. Therefore, developing new drugs and vaccines against scabies is an urgent problem to be solved. Exploring the interaction between *Ss*Galectins and host may provide new insights for developing novel anti-scabies drugs and prevention strategies.

Galectins are a family in the lectin superfamily that have 1 or 2 sugar recognition domains in their molecular structure. Galectin-1 (GAL-1) is a powerful anti-inflammatory and immunoregulatory factor that is involved in the pathophysiology of inflammatory disorders (Astorgues-Xerri et al., 2014). Galectin-3 (GAL-3) is an essential regulator of biological processes, and it has recently been found that it is closely related to the pathogenesis of host immune and inflammatory diseases (Nangia-Makker et al., 2000; Dumic et al., 2006). Galectin-2 (GAL-2) is mainly expressed in the gastrointestinal tract and can promote the healing of gastrointestinal epithelial wounds. Galectin-5 (GAL-5) is expressed in rat erythrocytes and may play a role in erythropoiesis (Barrès et al., 2010). In the study of parasite–host interactions, Galectins are engaged in a range of immune-related host life functions, including pathogen and host cell adhesion, activation of host innate and adaptive immunity, immune cell proliferation and death, and inflammatory modulation (Farhadi and Hudalla, 2016; Hafidi et al., 2021; Loghry et al., 2022). Macrophages are thought to play a crucial role in the innate immune response to scabies, and macrophage infiltration was observed in skin samples from several species of domestic and wild mammals (Martínez et al., 2020; Nwufoh et al., 2020). *S. scabiei* mite infection can elicit an innate immune response in the host. Although it has been shown that *S. scabiei* mites are able to inhibit host macrophages from participating in the inflammatory response by expressing macrophage migration inhibitory factors (Cote et al., 2013), skin biopsies from dogs with mange mites show the presence of macrophage infiltration within the skin (Nwufoh et al., 2020). Activation of macrophages leads to the production of prostaglandins, leukotrienes, and IL-31, which exacerbate host skin itching (Hashimoto et al., 2019; Arora et al., 2020). Galectins family is involved in the regulation of macrophages (Zangbede et al., 2018; Shi et al., 2020; Loghry et al., 2022). Thus, exploring the role of members of the Galectins family in the invasion of *S. scabiei* mites is of great significance for the study of the pathogenic mechanism of *S. scabiei* mites.

## Methods

### Mites and animals

*S. scabiei* mites and New Zealand white rabbits were provided by the Department of Parasitology at Sichuan Agricultural University. Mites (adults, nymphs, and larvae) were collected first, then live adult mites (male and female mites) were separated from the sample after morphological identification, and larvae and nymphs were left and collected together. Eggs are laid in the epidermis of the host skin and cannot be collected. Eight female Sprague-Dawley rats (6 weeks old, 150–200 g), 6-week-old female BALB/c mice (6 weeks old, 20–22 g), and 6-week-old male C57BL/6J mice (6 weeks old, 20–22 g) were purchased from SPF Biotechnology Company (Beijing).

### Macrophages in the scabies skin

Healthy and naturally infected New Zealand rabbit toe skins were fixed overnight with a 4% paraformaldehyde fixative (Biosharp, Guangzhou, China). The paraffin-embedded skin samples were sectioned to 5 μm, dried in an oven at 37°C for 24 h, deparaffinized, immersed in 0.1 M sodium citrate for 15 min for antigen retrieval, and washed three times with phosphate-buffered saline (PBS). The 5% goat serum was used to block at room temperature for 2 h, and then the primary antibody, F4/80 Rabbit pAb (1:200) (ABclonal, Wuhan, China), was incubated overnight at 4°C and washed three times with PBS, followed by the secondary antibody, FITC Goat Anti-Rabbit IgG (H+L) (1:400) (ABclonal, Wuhan, China), incubated for 45 min at room temperature. After washing four times, DAPI (Solarbio, Beijing, China) was used to incubate for 5 min at room temperature. Following mounting, tissue sections were examined and captured on a camera using a digital slice microscope [OLYMPUS, VS120-S6-W (BX61VS), Japan].

### Galectin mRNA levels in *S. scabiei* mites

Real-time quantitative polymerase chain reaction (RT-qPCR) analysis was performed to validate the mRNA expression levels of *Ss*GAL-1, *Ss*GAL-2, *Ss*GAL-3, and *Ss*GAL-5 in different developmental stages of scabies mites, and β-actin was used as a housekeeping gene. The collected mites of different stages were ground in a pre-cooled mortar. Total RNAs of larvae/nymph and adult mites were extracted using the RNAprep Pure Tissue Kit (Tiangen, Beijing, China) according to the manufacturer's instructions. RNA quality and quantity were assessed using the 1.5% agarose gel electrophoresis and the NanoDrop 2000 spectrophotometer. An amount of 1 μg of total RNA was reverse-transcribed to cDNA using the RevertAi™ First Strand cDNA Synthesis Kit (Thermo Fisher Scientific, Waltham, MA, USA) according to the manufacturer's instructions. The qPCR primers (Supplementary Table S1) were designed using the nucleic acid sequences of Galectins in NCBI (*Ss*GAL-1: JXLN01009935.1, *Ss*GAL-2: JXLN01010345.1, *Ss*GAL-3: JXLN01010568.1, *Ss*GAL-5: JXLN01013773.1) as templates, and the qPCR was carried out using the LightCycler480 System (Roche Diagnostics) after the primers were synthesized. The reaction mix (20 μl) includes SYBR Permix Ex TaqII (FOREGENE, Chengdu, China) 10 μl, upstream primer 0.8 μl, downstream primer 0.8 μl, cDNA 2 μl, and ddH_2_O 6.4 μl. The cycling conditions were as follows: an initial denaturation at 95 °C for 30 s, followed by 40 cycles at 95°C for 5 s and 60°C for 30 s, and then melting curve analysis at 95°C for 5 s, 60°C for 60 s, and 95°C for 1 s. qPCR was conducted in triplicate, and the relative gene expression levels were calculated using 2^−ΔΔCt^ method (Livak and Schmittgen, 2001).

### Expression and identification of *Ss*Galectins

Total RNA from *S. scabiei* mite (larvae+nymphs, and adults) was isolated and reverse-transcribed. Primers were created using the nucleic acid sequence of Galectins from NCBI as a guide (Supplementary Table S2), and PCR amplification was performed using scabies mite cDNA as a template. PCR products were separated and cloned into the PMD19-T vector (TaKaRa, Beijing, China) and subcloned into the pET32a (+) vector (Novagen, USA) using restriction sites (underlined in the above primers); then, the recombinant plasmids were sequenced (Sango, Shanghai, China). The identified recombinant plasmid was transformed into *E. coli* BL21 (DE3) cells (Invitrogen, USA), and 1 mM isopropyl β-D-1-thiogalactopyranoside was used to induce the expression of cultured cells. The recombinant protein was purified by chromatography with a Ni-NTA His-tag affinity kit (Bio-Rad, USA) according to the manufacturer's instructions. Recombinant protein was analyzed for purity by SDS-PAGE using a 12% gel and subsequently with a bicinchoninic acid protein assay kit (Pierce, USA) to estimate protein concentration. A Western blot was used to identify recombinant Galectins. Protein samples were boiled for 10 min in electrophoresis sample buffer before being separated by 12% SDS-PAGE. An electrophoretic transfer cell (Bio-Rad, USA) was used to transfer the protein onto a PVDF membrane, then the membranes were incubated with 5% skim milk for 2 h after washing three times with TBST, followed by 1:100 diluted serum samples [*S. scabiei* negative rabbit serum, positive rabbit serum, rat negative serum, and rat anti-*Ss*-GAL (1,2,3,5) IgG, respectively] at 4°C overnight after washing three times with TBST. Then, the membranes were incubated with horseradish peroxidase (HRP)-conjugated goat anti-rabbit IgG antibody or horseradish peroxidase (HRP)-conjugated goat anti-rat IgG antibody (1:3,000; Boster, China) for 2 h. After washing four times with TBST, the Enhanced HRP-DAB Chromogenic Substrate Kit (Tiangen, China) was used to visualize the protein signal according to the manufacturer's instructions.

### Immunohistochemical analysis of *Ss*Galectins

Paraffin sections of scabies mite skin and scabies mite bodies were produced in the same way as shown in macrophage localization, and the immunofluorescence procedure was performed as previously described. Eight healthy rats were prepared and randomly divided into four groups of two rats each. To prepare murine anti-r*Ss*Galectins-IgG, the purified r*Ss*GAL-1, r*Ss*GAL-2, r*Ss*GAL-3, and r*Ss*GAL-5 were used as antigens for immunization for each group by subcutaneous injection, respectively. Purified rat anti-r*Ss*GAL-1-IgG, r*Ss*GAL-2-IgG, r*Ss*GAL-3-IgG, r*Ss*GAL-5-IgG, and rat negative serum were used as primary antibodies (diluted in 1:400), and FITC Goat Anti-Rat IgG (H+L) (1:400) (ABclonal, Wuhan, China) was used as secondary antibody to conduct immunofluorescence localization experiments on scabies skin and scabies mites, respectively, to determine the distribution of *Ss*GAL-1, *Ss*GAL-2, *Ss*GAL-3, and *Ss*GAL-5 in scabies mites and secretion in scabies skin. H&E staining was done as a control to make the fluorescent immunohistochemistry results more understandable.

### Establishment of AD model and stimulation of recombinant protein

On day 0, a 3 cm by 3 cm portion of the mice's backs had their hair removed. Then, 200 μl of 1% 1-chloro-2,4-dinitrobenzene (DNCB) (olive oil:acetone = 4:1) (Sigma-Aldrich, St. Louis, MO) was evenly applied to the region that has been shaved on days 1–3. An amount of 100 μg of dialyzed and purified r*Ss*GAL-1, r*Ss*GAL-2, r*Ss*GAL-3, and r*Ss*GAL-5 was administered subcutaneously at the same place on days 8 through 14. The control group received the same dose of tacrolimus (MedChemExpress, USA), histamine (VETEC, USA), recombinant pET32a protein, and PBS injections, respectively (10 BALB/c mice for each group), and on days 8, 11, and 14, 4% SDS was applied for 2 h, and then 0.5% DNCB was applied to keep the dermatitis condition. Tacrolimus (Tac) is a macrolide immunosuppressant with anti-inflammatory, antipruritic, and immunomodulatory properties that are extensively used in the treatment of AD skin disorders. Histamine (His) can elicit allergy symptoms such as skin irritation, itching, erythema, and wheal and can be utilized as a positive control in AD models. The tissue samples from the skin lesions were collected on the 15th day, the skin thickness was measured with a micrometer (DeQing, Zhejiang, China), and the samples were kept in a −80°C refrigerator or in a 4% paraformaldehyde fixative solution.

### H&E and macrophages in the AD model

Paraffin sections of mouse AD model skin were prepared as previously described. Stained with hematoxylin and eosin (H&E) to determine the thickening and cellular infiltration of the skin. Furthermore, the immunofluorescence procedure was performed in the same way as shown in macrophage localization. F4/80 Rabbit pAb (1:200) (ABclonal, Wuhan, China) was employed as the primary antibody and FITC Goat Anti-Rabbit IgG (H+L) (1:400) (ABclonal, Wuhan, China) as the secondary antibody to observe macrophage infiltration in experimental mice. A digital section microscope was used to monitor and photograph both H&E and F4/80 practical sections.

### Preparation and culture of bone marrow-derived macrophages from mice

The collection of bone marrow cells and differentiation into bone marrow-derived macrophages were performed according to Toda et al. (2021). Cells were resuspended in 400 μl PBS and evenly divided into four tubes. CD11b^+^ (Elabscience, Wuhan, China) and F4/80^+^ (BioLegend, Beijing, China) fluorescent dyes were used for staining at 4°C for 30 min. Before flow cytometry, cells were resuspended in 400 μl PBS. The Kaluza 2.1 program was used to examine the data. More than 85% of the cells bearing CD11b^+^ and F4/80^+^ double-labeled antibodies could be recognized as macrophages.

### Effects of recombinant *Ss*Galectins on BMDMs

The working concentration range of recombinant Galectins (0, 0.1, 1, 10, 50, and 100 μg) was established using the CCK-8 (Solarbio, Beijing, China) assay. Based on the results of the CCK-8 cytotoxicity assay, 20 μg r*Ss*GAL-1, r*Ss*GAL-2, r*Ss*GAL-3, and r*Ss*GAL-5 proteins were added to each well and incubated for 24 h, while 20 μg of pET32a protein and 20 μl PBS were added to each well and incubated for 24 h as a blank control group. A volume of 1 ml of BMDM suspension was added to each well of a 6-well plate (the number of cells was approximately 10^*^6), and every group received three samples that were identical. Cell supernatant was gathered and kept at −80°C in storage. The cells were washed two times with PBS, and the protein was immediately removed in preparation for further research.

### qPCR analysis of inflammatory factors

The impact of recombinant galectins on IL-4, IL-12, TGF-β, IL-6, IL-10, IL-1β, inducible nitric oxide synthase (iNOS), and Arg1 sections was examined using real-time PCR analysis (Na, 2019; Feng et al., 2020). The BMDM cells were collected, the total RNA was extracted as directed, and then the RNA obtained was reverse-transcribed into cDNA. The reaction mixture and cycling conditions were maintained as described previously. The qPCR was carried out using the LightCycler480 System (Roche Diagnostics) after primers were synthesized according to the reported primer sequences (Supplementary Table S3). The β-actin gene was simultaneously used as a reference to normalize the data. qPCR was conducted in triplicate, and the relative gene expression levels were calculated using the 2^−ΔΔCt^ method (Livak and Schmittgen, 2001).

### Enzyme-linked immunosorbent assay

The above cell supernatant was collected, and then, IL-1β, IL-6, TGF-β, IL-10, iNOS, and Arg1 were detected according to the ELISA kit instructions (4A BIOTECH/CUSABIO, China).

### Statistical analysis

Statistics are expressed as mean ± standard deviation (X ± SD, *n* = 3) and were determined using one-way analysis of variance (ANOVA) using the GraphPad Prism version 8.0 software (GraphPad Software, USA). Probabilities (p) less than 0.05, 0.01, or 0.001 were considered significant (^*^p < 0.05, ^**^p < 0.01, or ^***^p < 0.001), and a *p*-value of above 0.05 means no significant difference (*p* > 0.05). Graphs were created using the GraphPad Prism version 8.0 software.

## Results

### Macrophages in the skin of scabies

**Figure 1:**
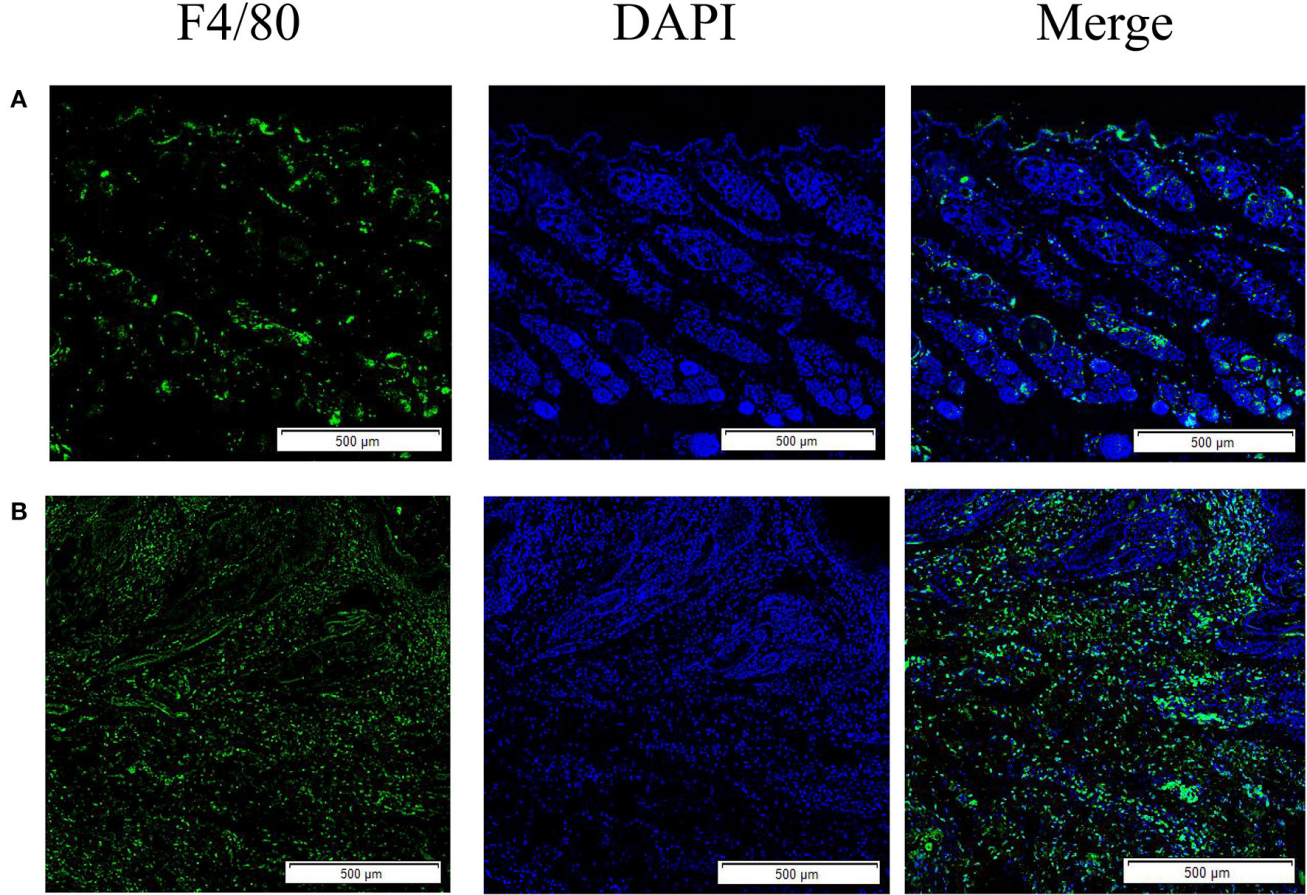
Skin macrophage immunofluorescence staining. **(A)** Immunofluorescence staining of healthy rabbit skin; **(B)** immunofluorescence staining of skin tissue infection with *S. scabiei*. Scale bar = 500 μm.

The results showed that the green fluorescence intensity of scabies mite skin was higher than that of healthy skin, indicating that scabies mite disease can cause an increased infiltration of macrophages in rabbit skin. F4/80 was used as a macrophage surface marker to label macrophages for immunofluorescence staining (Figure 1).

### mRNA expression profiles of Galectins

**Figure 2:**
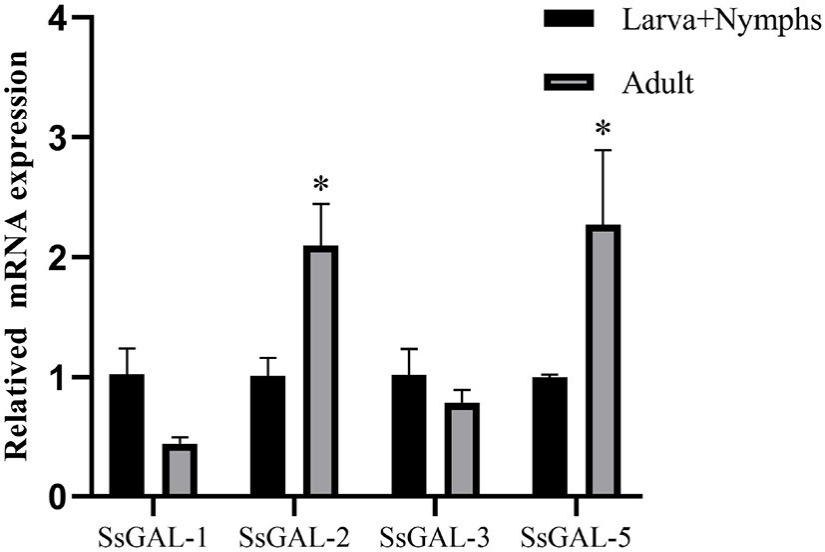
Transcriptional levels of *Ss*Galectins in different developmental stages. Data are represented as the mean ± standard deviation (M ± SD) of three replicates per groups. A one-way ANOVA analysis was used to analyze the variances between the groups. “*” is the secretion level of the treatment group compared with the control group (*p < 0.05).

The *Ss*GAL-1, *Ss*GAL-2, *Ss*GAL-3, and *Ss*GAL-5 were expressed in adult mites and in the mixed sample of larvae and nymphs. The expression levels of *Ss*GAL-2 and *Ss*GAL-5 in adult mites were higher than in mixed samples; however, the expression levels of *Ss*GAL-1 and *Ss*GAL-3 in adult mites were slightly lower than those in mixed samples (Figure 2).

### Expression and identification of r*Ss*Galectins

**Figure 3:**
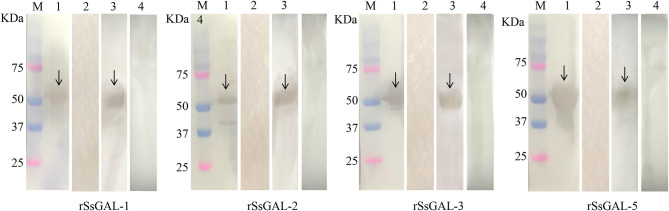
r*Ss*Galectins immunogenicity analysis. M: Protein marker; 1: Rat polyclonal antibody reacts with recombinant protein; 2: Rat negative serum reacts with recombinant protein; 3: Rabbit sera infected with scabies mites react with recombinant protein; 4: Healthy rabbit serum reacts with recombinant protein.

The *Ss*GAL-1, *Ss*GAL-2, *Ss*GAL-3, and *Ss*GAL-5 genes were successfully cloned from the cDNA of the mixed sample (larvae, nymph, and adult mites) with specific primers. The size was all around 1,000 bp (1,131, 1,080, 1,008, and 1,020 bp, respectively). The r*Ss*GAL-1 and r*Ss*GAL-5 presented as soluble proteins, while the r*Ss*GAL-2 and r*Ss*GAL-3 presented in inclusion bodies. SDS-PAGE analysis revealed that galectins were purified as clear and single bands at locations 59, 57, 54, and 55 kDa, respectively (the His-tag protein of the expression vector was 18 kDa). Four r*Ss*Galectins were specifically recognized by *S. scabiei*-positive rabbit serum and anti-*Ss*-GAL (1,2,3,5) IgG, but no band was observed after incubation with *S. scabiei*-naive rabbit serum (Figure 3).

### Immunohistochemical analysis of *Ss*Galectins

**Figure 4:**
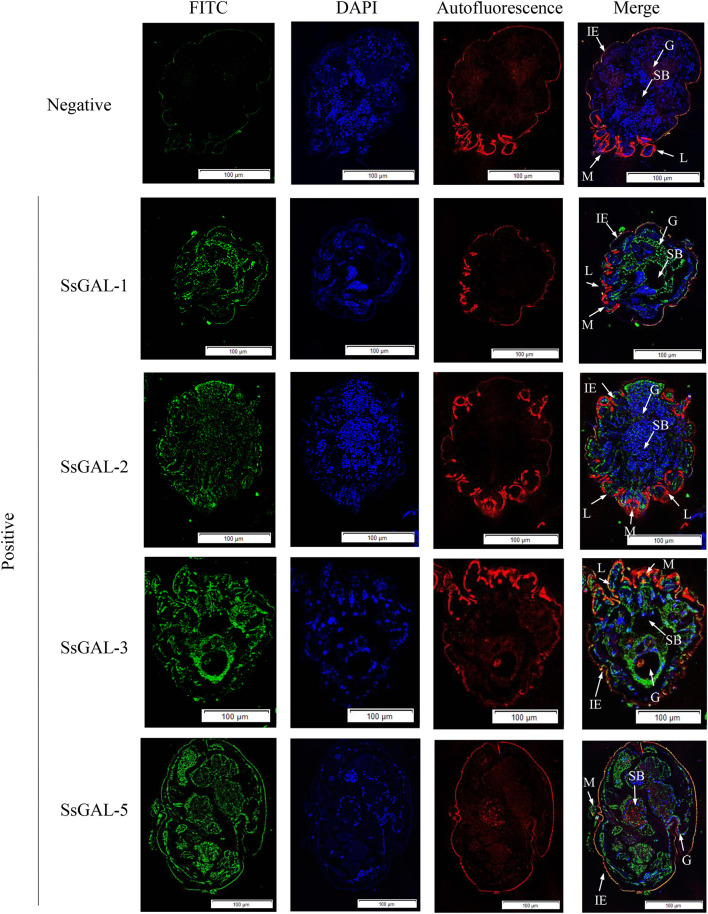
Immunofluorescence of *Ss*Galectins. Positive: Incubated with rat anti-r*Ss*Galectins antibody as the primary antibody; Negative: Incubate with rat negative serum as the primary antibody. M, mouthpart; L, leg; G, gut; SB, stomach blocks; IE, epidermal integument. Green fluorescence indicates F4/80 as an antibody to recognize macrophages, blue fluorescence indicates DAPI-labeled nuclei, and red fluorescence is skin autofluorescence. Scale bar = 100 μm.

**Figure 5:**
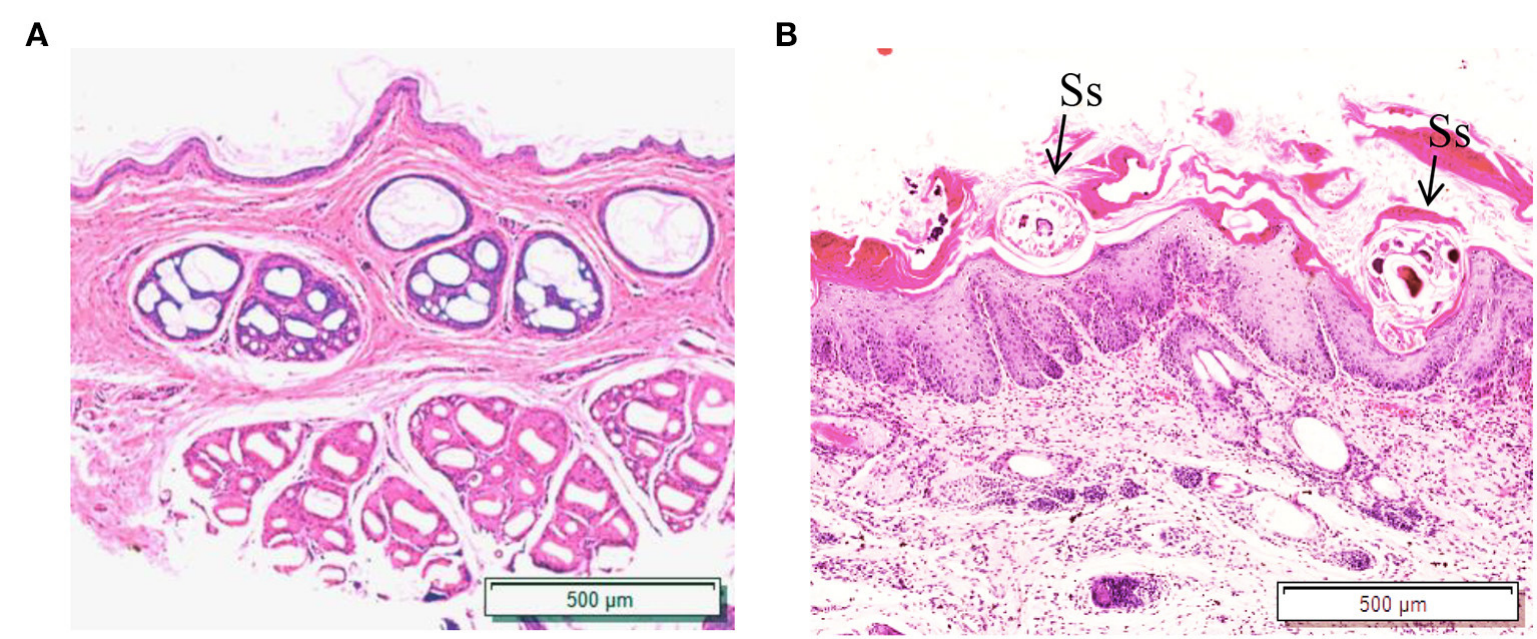
Hematoxylin and eosin (HandE) staining of rabbit skin. **(A)** Healthy skin; **(B)** Scabies skin. The arrows point to the *S. scabiei*. Scale bar = 500 μm.

**Figure 6:**
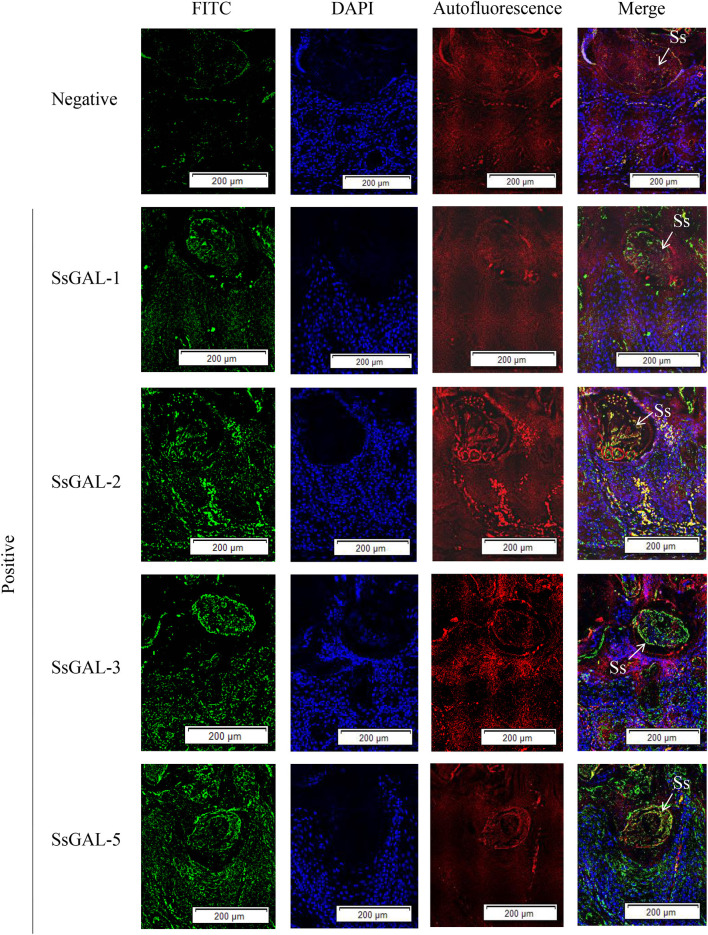
Immunofluorescence of *Ss*Galectins in the skin of rabbits infected with scabies. Positive: Incubated with rat anti-r*Ss*Galectins antibody as the primary antibody, Negative: Incubated with negative rat serum as the primary antibody. Green fluorescence indicates F4/80-labeled macrophages, blue fluorescence indicates DAPI-labeled nuclei, and red fluorescence is skin autofluorescence. The arrows point to the *S. scabiei*. Scale bar = 500 μm.

No green fluorescence was detected in the scabies skin or scabies mites using negative serum from rats. On the body surface of scabies mites, *Ss*GAL-1 and *Ss*GAL-2 were discovered to include the mouthpiece, limbs, and epidermis, whereas *Ss*GAL-3 and *Ss*GAL-5 were widely distributed within and on the bodies of scabies mites and also included the gut and stomach (Figure 4). Furthermore, green fluorescence was discovered not only on the mites but also on the scabies skin (Figures 5, 6).

### Clinical symptoms of AD model mice

**Figure 7:**
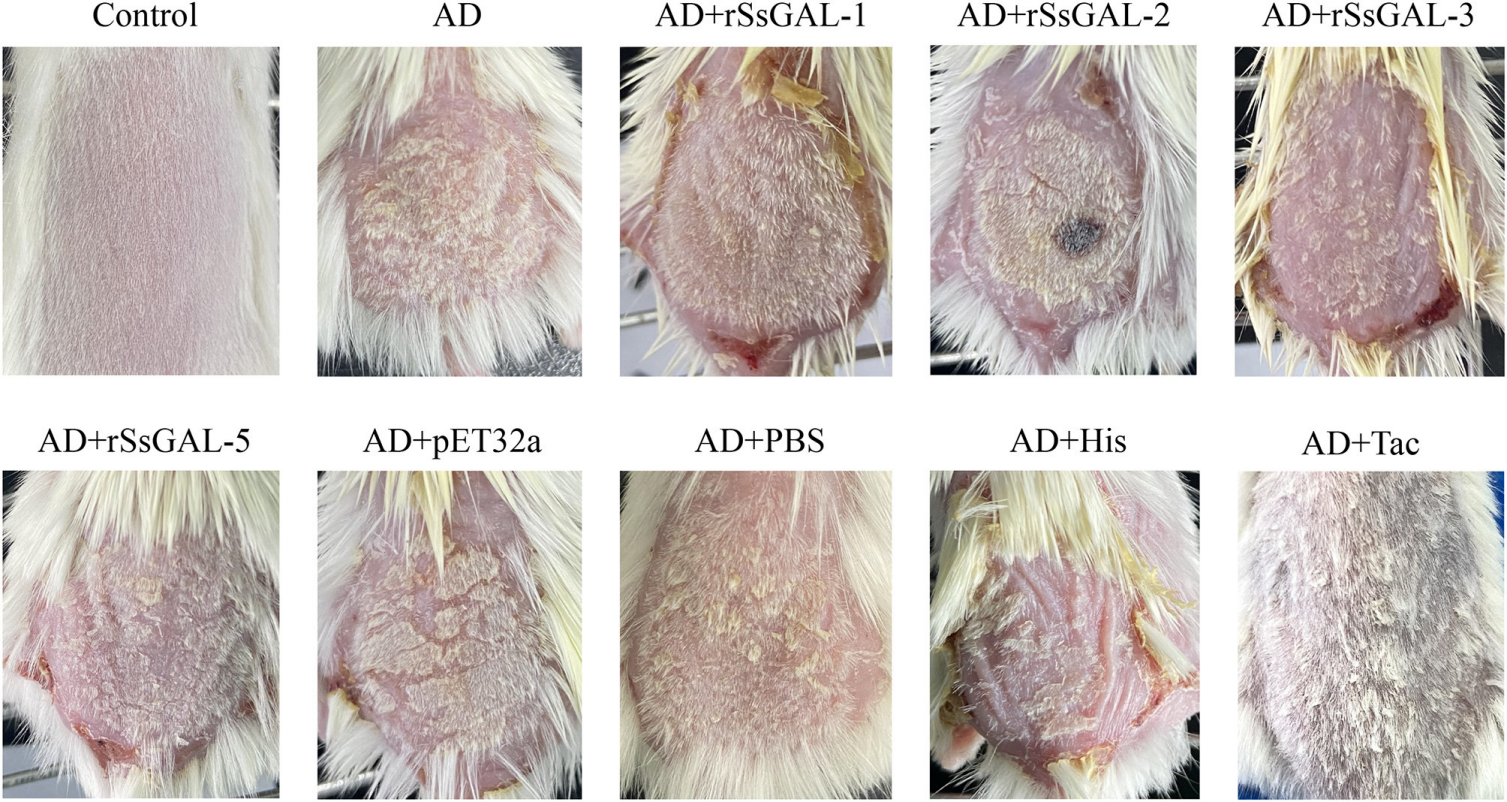
Photographs of mice's back skin from each group on day 15.

On the 5th day, after 3 days of continuous application of 1% DNCB, erythema, edema, and a minor number of scabs occurred on the back skin of mice. With continued application of 0.5% DNCB, the mice exhibited severe scratching, dry skin, scab thickness, erosion, and bleeding. There was no significant difference in clinical symptoms between the r*Ss*GAL-1 and r*Ss*GAL-5 protein groups compared to the AD group after 8–14 days of recombinant protein stimulation. Compared to the AD group, the r*Ss*GAL-2 and r*Ss*GAL-3 protein groups experienced more severe crusting and exudation on the skin surface, similar to histamine group symptoms. The Tacrolimus group had reduced erythema on the skin's surface, no significant scabs, and more hair. The pET32a (+) group exhibited slightly thicker crusts than the AD group, but there was no difference in symptoms between the PBS and AD groups (Figure 7).

### H&E of mice skin

**Figure 8:**
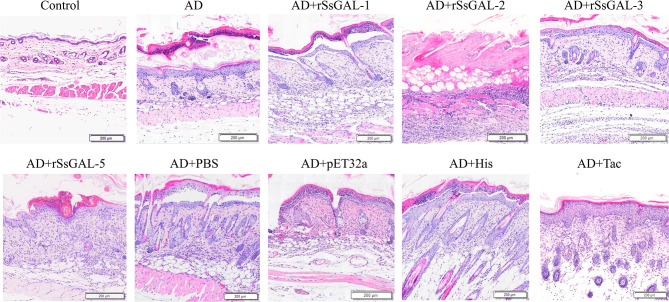
HandE staining of back skin sections. Scale bar = 200 μm.

It was clear that the mouse AD model was developed successfully because the stratum corneum of the back skin of the mice in the AD group had been damaged, showing hyperkeratosis, parakeratosis, thickness of the spinous layer, and infiltration of inflammatory cells in the dermis. Furthermore, inflammatory cell infiltration in the dorsal skin of mice was higher in the r*Ss*GAL-1, r*Ss*GAL-2, r*Ss*GAL-3, and r*Ss*GAL-5 groups than in the AD group, and the pathological alterations in the skin tissue were more severe. In the r*Ss*GAL-2 group, some necrotic subcutaneous tissues were discovered. The His group had the most severe pathological abnormalities as compared to the positive control group. In contrast, the Tac group had typical stratum corneum structure, thickening of the spinous layer, and spinous layer edema. Inflammatory cells infiltrated more in the pET32a group than in the AD group, but there was no significant difference in the PBS group (Figure 8).

### Immunofluorescence of mice skin

**Figure 9:**
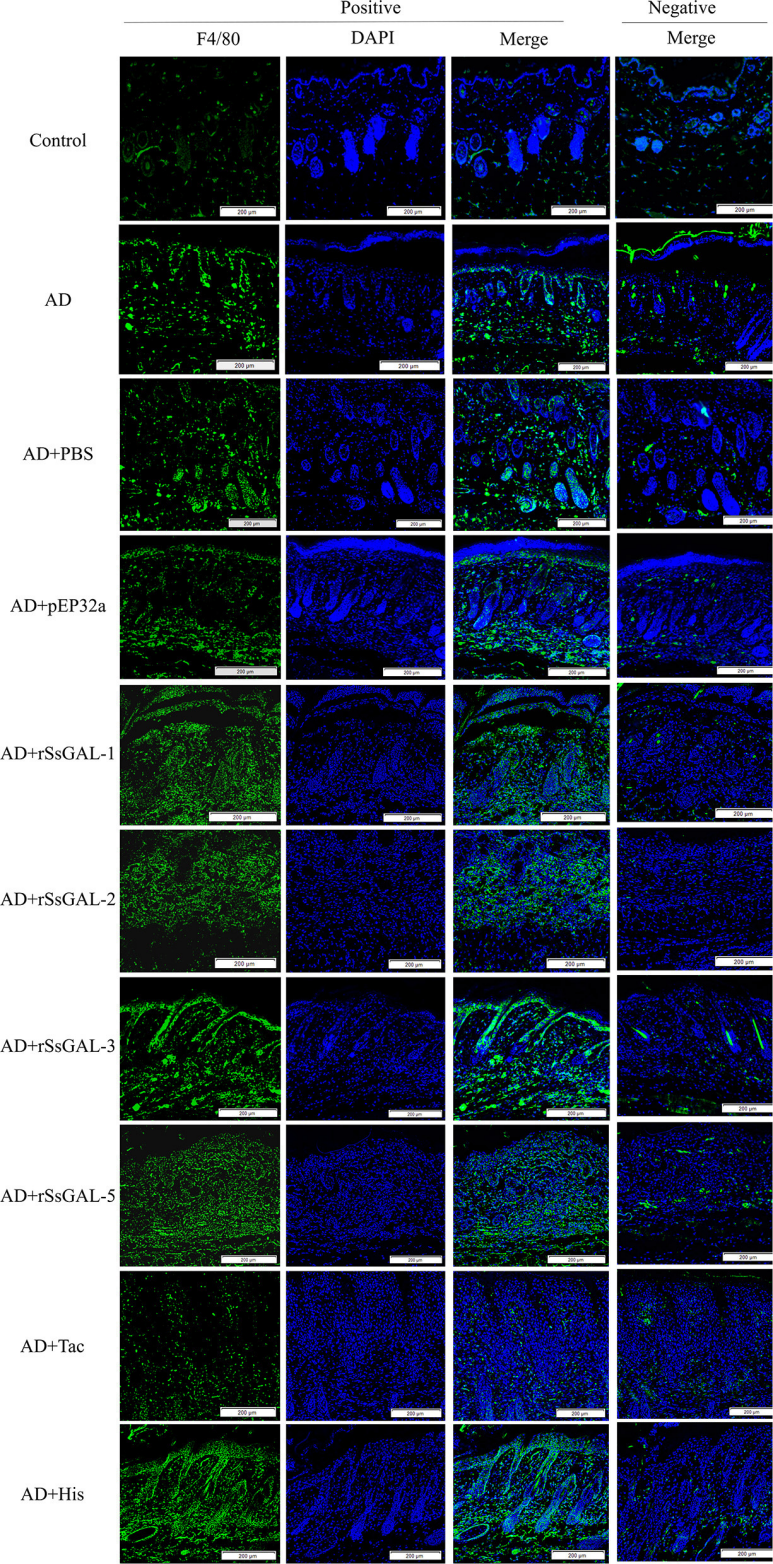
Immunofluorescence staining of skin lesions. Green fluorescence indicates F4/80-labeled macrophages and blue fluorescence indicates DAPI-labeled nuclei. Scale bar = 200 μm.

The green fluorescence of the AD group was higher than that of the healthy group. The fluorescence intensity of the r*Ss*GAL-1, r*Ss*GAL-2, r*Ss*GAL-3, and r*Ss*GAL-5 groups rose compared to the AD group, particularly the r*Ss*GAL-3 group, which was similar to the His group. The Tac group's fluorescence intensity was reduced; there was no significant difference between the PBS group, the pET32a group, and the AD group (Figure 9).

### Flow cytometry and CCK-8 assay

**Figure 10:**
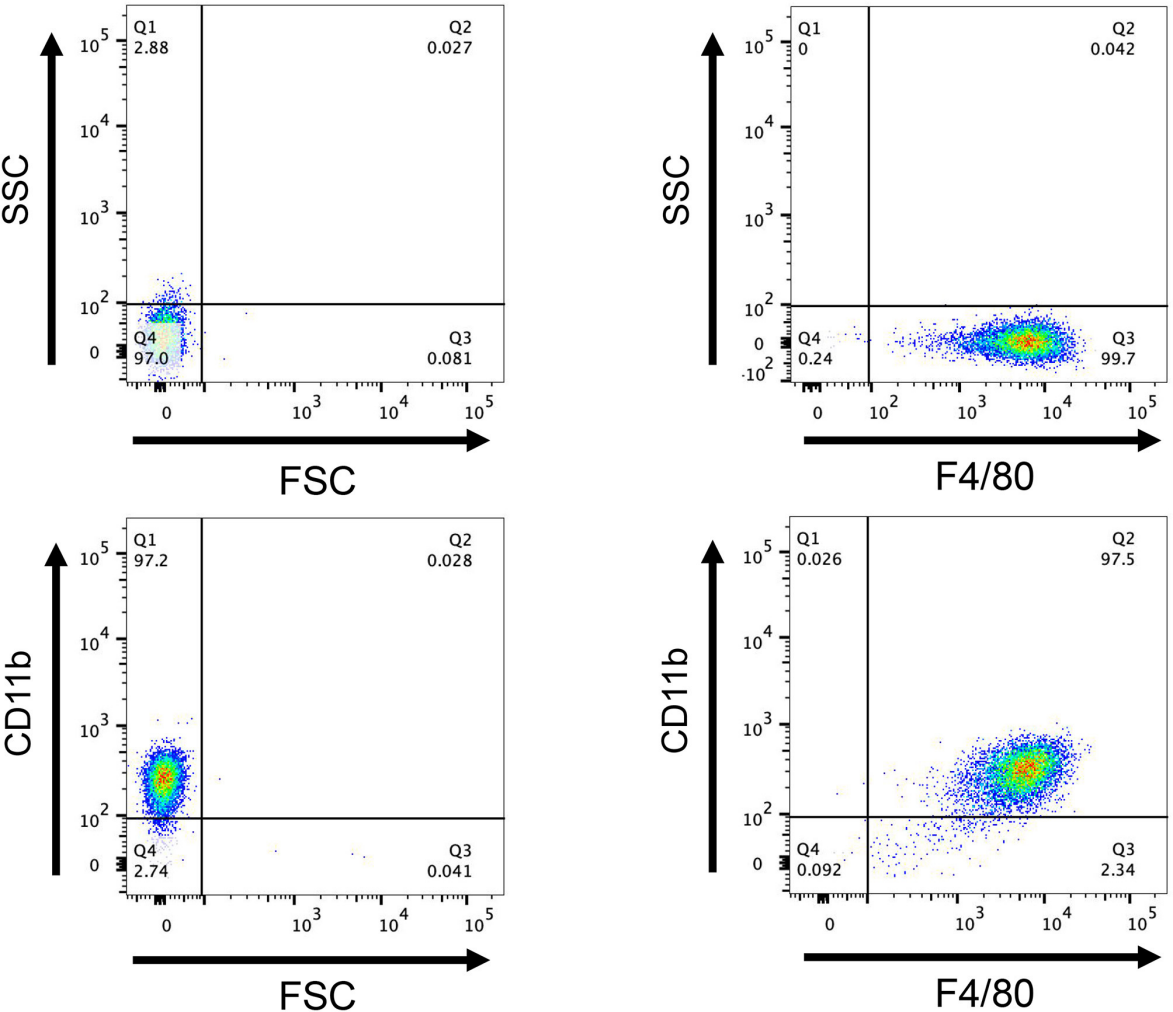
Flow cytometry detection of macrophages. After induction of mouse bone marrow-derived cells for 7 days, the expression of CD11b^+^ and F4/80^+^, a marker molecule for macrophages, was detected by flow cytometry. FCS, Reactive cell volume; SSC, Reflects cell granularity.

Flow cytometry revealed that 99.7% of F4/80^+^ cells were present, 97.2% of CD11b^+^ cells were present, and 97% of F4/80^+^ CD11b^+^ cells were present (Figure 10), showing that this approach successfully converted bone marrow cells into BMDM. The CCK8 test findings indicated that the cell survival rates of r*Ss*GAL-1, r*Ss*GAL-2, r*Ss*GAL-3, r*Ss*GAL-5, and recombinant pET32a proteins were all over 90% at 0.1–100 μg, showing that the above recombinant proteins were effective at 0.1–100 μg. The viability of BMDM has no influence, and further tests can be conducted within this dose range.

### qPCR of inflammatory factors

**Figure 11:**
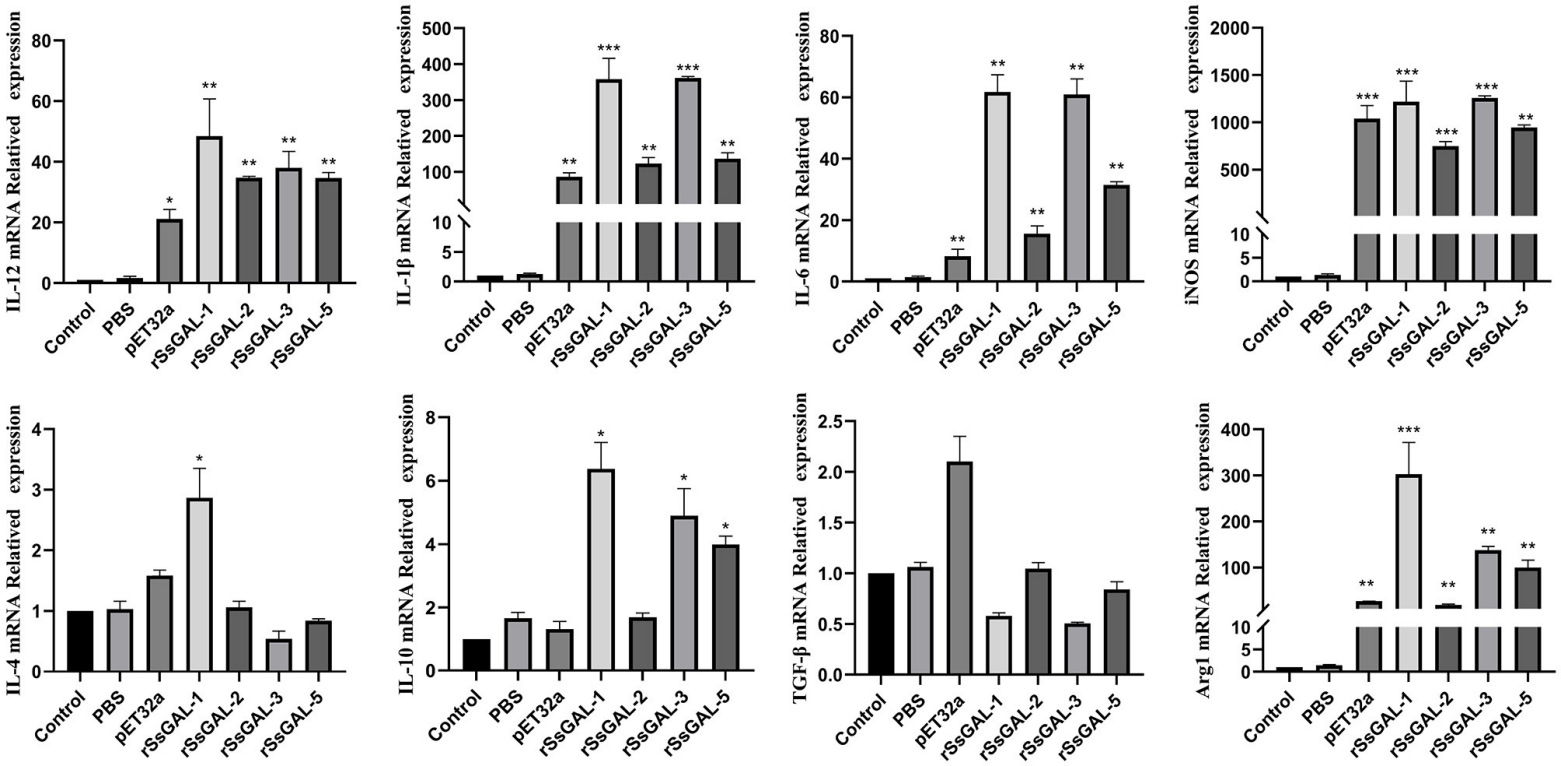
Relative transcript levels of inflammatory-related factors in BMDM. Data are represented as the mean ± standard deviation (M ± SD) of three replicates per groups. A one-way ANOVA analysis was used to analyze the variances between the groups. “*” is the secretion level of the treatment group compared with the control group (*p < 0.05, **p < 0.01, ***p < 0.001).

The mRNA transcription levels of IL-1β, IL-6, IL-12, iNOS, and Arg1 were higher in the r*Ss*GAL-1, r*Ss*GAL-2, r*Ss*GAL-3, r*Ss*GAL-5, and recombinant pET32a groups than in the control group (*p* < 0.01). Simultaneously, the levels of IL-4 and IL-10 mRNA transcription were increased (*p* < 0.05) in the r*Ss*GAL-1 group, while the transcription of IL-10 mRNA was increased (*p* < 0.05) in the r*Ss*GAL-3 and r*Ss*GAL-5 groups. The PBS group and the blank control group had no significant change in the relative transcription levels of inflammatory-related factors (*p* > 0.05) (Figure 11).

### ELISA of inflammatory factors

**Figure 12:**
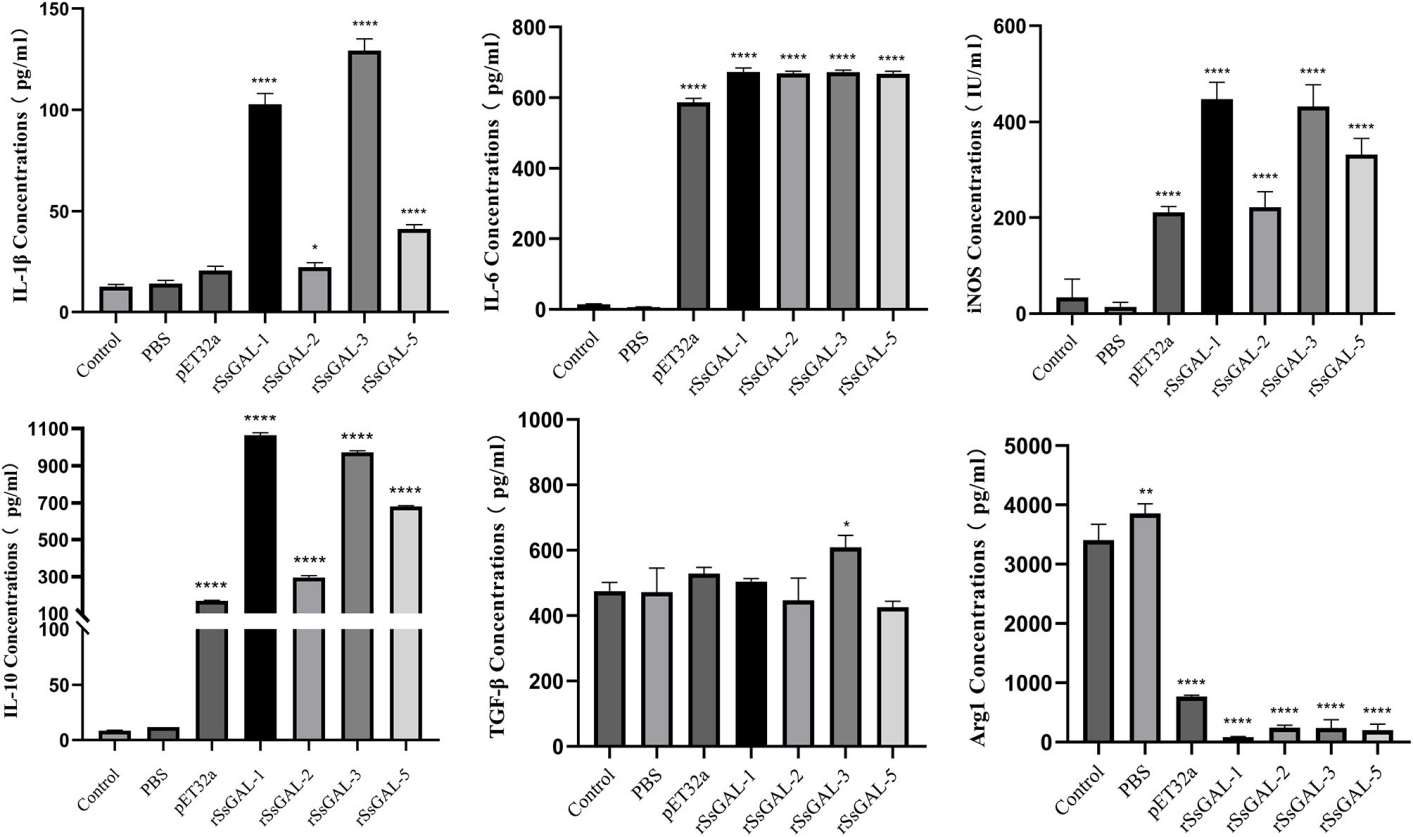
Secretion levels of inflammatory-related factors in BMDM. Data are represented as the mean ± standard deviation (M ± SD) of three replicates per groups. A one-way ANOVA analysis was used to analyze the variances between the groups, “*” is the secretion level of the treatment group compared with the control group (*p < 0.05, **p < 0.01, ****p < 0.0001).

ELISA detection of inflammatory cytokines in BMDM cell supernatant revealed that r*Ss*GAL-1, r*Ss*GAL-2, r*Ss*GAL-3, and r*Ss*GAL-5 increased IL-10, IL-1β, IL-6, and iNOS (*p* < 0.01), but Arg1 reduced (*p* < 0.01). TGF-β increased in the r*Ss*GAL-3 group, but there was no significant difference between the r*Ss*GAL-1, r*Ss*GAL-2, and r*Ss*GAL-5 groups (*p* > 0.05). Meanwhile, IL-10, IL-1β, and iNOS levels were higher in the r*Ss*GAL-1 and r*Ss*GAL-3 groups than in the r*Ss*GAL-2 and r*Ss*GAL-5 groups. Only Arg1 rose considerably in the PBS group (*p* > 0.05) (Figure 12).

## Discussion

The parasite body surface contains a variety of complex carbohydrates, including glycolipids, glycoproteins, and glycosylated phosphatidylinositol glycolipids, and these glycoconjugates play an important role in host invasion. Specific interactions between parasite glycoconjugates and host galectins are required for pathogen recognition. Glycoconjugates cause pro-inflammatory effects in the host, antigen presentation, and innate immune response (Mascanfroni et al., 2011). Galectins have a high affinity for β-galactoside and are involved in various physiological and pathological processes, such as cell adhesion, apoptosis, and inflammatory response.

F4/80 is a surface marker of macrophages and shows green fluorescence, and the green fluorescence was stronger in the scabies skin, demonstrating that *S. scabiei* invasion causes macrophage infiltration in the host skin. *S. scabiei* has a life cycle that includes eggs, larvae, nymphs, and adults. Eggs are typically found in the meandering tunnels of the skin epidermis. By microscopy, the larvae are tiny and difficult to distinguish from the nymphs. As a result, the larvae and nymphs are gathered together, while the adult mites are collected separately in this study. *Ss*GAL-1, *Ss*GAL-2, *Ss*GAL-3, and *Ss*GAL-5 were expressed at each developmental stage of *S. scabiei*, with *Ss*GAL-2 and *Ss*GAL-5 having higher transcript levels in adults, indicating that *Ss*Galectins play a crucial role in the development of *S. scabiei*, especially during scabies mite pathogenesis. It has been observed that the transcriptional level of galectins in *Haemonchus contortus* is discovered in distinct developmental stages, and *Hco*GAL-1 is significantly expressed in each developmental stage, *Hco*GAL-3 is exclusively expressed in adults, and *Hco*GAL-4 is expressed in L3 larvae (Greenhalgh et al., 2000). It implies that different galectins may participate in the parasite's life activities at various stages.

Based on the genomic sequence of *S. scabiei*, we cloned, expressed, and discovered Galectins. The Western blot analysis revealed that r*Ss*Galectins were specifically recognized by *S. scabiei*-positive rabbit serum and anti-*Ss*-GAL (1,2,3,5) IgG. Immunohistochemical analysis of *Ss*Galectins in scabies skin revealed that *Ss*Galectins could be secreted and diffused into the host skin, confirming that r*Ss*Galectins were an essential component of ES antigens. *Ss*Galectins may play a significant role in parasite-induced inflammation in the host. As a result, understanding the mechanism of scabies mites on host inflammation is the primary goal for scabies mite prevention and therapy.

By generating different cytokines and growth factors, macrophages execute antigen presentation, phagocytosis, and immunological control (Fujiwara and Kobayashi, 2005; Chazaud, 2020). Macrophages are crucial for the initiation, continuation, and resolution of inflammation (Fujiwara and Kobayashi, 2005). Macrophages can secrete some pro-inflammatory factors to exacerbate inflammation and microbial clearance and can also produce anti-inflammatory factors to inhibit inflammation (Hofmann et al., 2010; Mounsey et al., 2015). This is due to a process called macrophage polarization, which allows macrophages to divide into cells with different functions in response to various stimuli. According to classical immunology, they are categorized as either classically activated macrophages (M1 macrophages) or alternatively activated macrophages (M2 macrophages), depending on the type of stimulus and the polarized cell phenotype. M1 macrophages primarily secrete pro-inflammatory factors, perform immune defense and pro-inflammatory functions, and could significantly increase iNOS and TNF-α levels. Thus, IL-6, IL-12, iNOS, and TNF-α can be used as molecular markers to identify M1 macrophages. On the contrary, M2 macrophages primarily release anti-inflammatory factors and exhibit anti-inflammatory effects, and their marker molecules include IL-10, Arginase 1 (Arg1), and TGF-β (Funes et al., 2018; Reichel et al., 2019). Macrophage polarization is a dynamic process, and under specific conditions, M1 and M2 types can be interconverted (Yang et al., 2021). Early in local inflammation, the afflicted macrophages exhibit the M1 type, which has a strong potential to deliver antigens. On the contrary, persistent M1-type macrophages cause excessive tissue damage and impair wound healing (Hu et al., 2012). When the inflammation is severe enough to interfere with the body's normal physiological operations, M2 macrophages expand, removing debris and apoptotic cells with great phagocytic capabilities and secreting anti-inflammatory substances to assist wound healing.

Scabies mite migratory activity, excreta production, and scabies mite breakdown products after death can induce allergic dermatitis in the host (Gazi et al., 2022). Scabies is classified into two types of clinical manifestations, namely, ordinary scabies (OS), and crusted scabies (CS). OS is produced mainly by a limited number of mites and appears as papules, erythema, and allergic skin responses with intense itching. CS has a significant number of mites and eggs in the skin epidermis, and the epidermis is thick and scaly (Arlian and Morgan, 2017; Stienstra et al., 2019). Atopic dermatitis (AD) is a chronic inflammatory skin condition marked by intense itching, recurring eczema, erythema, and dry skin. Currently, AD models are often used in fundamental research on allergic dermatitis. Many studies have employed 1-chloro-2,4-dinitroenzene (DNCB) to induce a persistent clinical AD model (Lee et al., 2022). Tacrolimus is a macrolide immunosuppressant that is widely used to treat AD. It has anti-inflammatory, antipruritic, and immunomodulatory characteristics (Bajgai et al., 2017). Histamine (His) can elicit allergy symptoms such as skin irritation, itching, erythema, and wheal, and it can be utilized as a positive control (Tachibana et al., 1990; Kim et al., 2013). In addition, an AD mouse model that is equivalent to the etiology and clinical indications of scabies was selected for future study due to a lack of secondary antibodies for rabbit cytokines.

It was found that the host GAL-3 and GAL-9 can attach to the parasite and cause it to bind to macrophages by detecting and binding to the β-galactosyl epitope on the lipopolysaccharide on the surface of Leishmania, potentially assisting the parasite in invading the body and causing infection (Pelletier et al., 2003; Turner et al., 2008). By attaching to annexin (Annexin A2) and activating JNK in the apoptotic signaling cascade, *Angiostrongylus cantonensis* GAL-1 can cause macrophage apoptosis (Shi et al., 2020). Exogenous galectins have been demonstrated in several disease models to have either anti-inflammatory or pro-inflammatory effects (Bastón et al., 2014; Martínez-Bosch et al., 2014). In this study, an AD model was constructed to investigate the involvement of r*Ss*Galectins in scabies. Observing changes in skin lesions and macrophage infiltration suggested that r*Ss*Galectin was implicated in the incidence and progression of scabies, primarily through pro-inflammatory effects. Additionally, alterations in BMDM inflammation-related variables following the stimulation with r*Ss*Galectins were discovered, and the impact of r*Ss*Galectins on macrophages was studied using cultured BMDM cells.

The mRNA transcription levels of IL-1β, IL-6, IL-12, iNOS, and Arg1 were significantly higher in the r*Ss*GAL-1, r*Ss*GAL-2, r*Ss*GAL-3, r*Ss*GAL-5, and pET32a (+) groups compared to those in the blank control group, according to qPCR data, indicating that the relative transcription level of M1 polarization marker factors is mainly increased by r*Ss*GAL-1, r*Ss*GAL-2, r*Ss*GAL-3, and r*Ss*GAL-5. It is hypothesized that r*Ss*Galectins triggers the polarization of BMDM to the M1 type, which enhances the inflammatory response. Among these, the transcription levels of both iNOS and Arg1 increased at the same time, with iNOS increasing significantly more than Arg1. It is possible that the r*Ss*Galectins family can drive BMDM polarization to the M1 and M2 types. However, M1-type polarization is predominant, with only a few cells polarized to the M2 type. According to ELISA data, r*Ss*GAL-1, r*Ss*GAL-2, r*Ss*GAL-3, and r*Ss*GAL-5 increased IL-10, IL-1β, IL-6, and iNOS inflammatory components while decreasing Arg1 levels. It indicated that the inflammatory response might be induced by r*Ss*GAL-1, r*Ss*GAL-2, r*Ss*GAL-3, and r*Ss*GAL-5 by encouraging BMDM cells to secrete the M1 polarization regulators IL-1β, IL-6, and iNOS.

IL-10 is an endogenous anti-inflammatory molecule that also acts as a maker for M2 macrophages (Gao et al., 2014). However, when BMDM was mainly polarized to M1, the expression level of IL-10 was dramatically raised in this research. IL-10 is an endogenous anti-inflammatory substance that also serves as a marker for M2-type macrophage polarization (Gao et al., 2014). M2 macrophages are classified into four subtypes, namely, M2a, M2b, M2c, and M2d. Although all M2 isoforms express IL-10, the cytokines produced and their roles vary. In this study, IL-10, IL-1β, and IL-6 expression levels increased when r*Ss*Galectins acted on BMDM; however, TGF-β did not change significantly. It is possible that the polarization type of a small number of M2 macrophages mediated by rSsGalectins is M2b. M2b macrophages, also known as “type II” macrophages, are polarized states between M1 and M2a, with both pro-inflammatory and anti-inflammatory effects involved in the regulation of late cellular inflammation (Wang et al., 2019; Gharavi et al., 2022). This indicates that macrophages are in the process of changing from the MI type to the M2 type. Arg1 is essential in the anti-infection mechanism of several parasitic diseases. If the host is infected with *Schistosoma mansoni*, Arg1 can effectively suppress Th2-mediated fibrosis, Th1/Th17-mediated intestinal damage, iNOS production, and endotoxemia (Pesce et al., 2009; Herbert et al., 2010). The mRNA transcription level of Arg1 was dramatically enhanced after r*Ss*Galectins were treated with BMDM in this investigation; however, the protein level was lowered. This could be connected to microRNAs' post-transcriptional control of macrophages. MicroRNAs are tiny noncoding RNAs that bind to the 3′ untranslated region of mRNA and govern post-transcriptional gene silencing (Iwakawa and Tomari, 2015). MicroRNAs have been implicated in the regulation of macrophage polarization, inflammation, and cancer progression (Li et al., 2018). For instance, miR-155 inhibits M2-type polarization and decreases Arg1 by targeting C/EBP, promoting the production of M1-type cell-related marker factors, such as TNF-α, IL-1β, and IL-6, and increasing the levels of mRNA and post-transcriptional translation of Arg1 (Arranz et al., 2012). In conclusion, by controlling macrophage polarization, r*Ss*Galectins play a role in the occurrence and progression of host inflammation. r*Ss*GAL-1, r*Ss*GAL-2, r*Ss*GAL-3, and r*Ss*GAL-5 all contributed to the pathogenic process of scabies by fostering inflammation in the host. This study offers new information on the immune relationship between the scabies mite and the host as well as a different approach to the investigation of the scabies pathogenic mechanism.

## Conclusion

The *Ss*Galectins are involved in the pathogenic process of *S. scabiei* by regulating the polarization of host macrophages to M1 type when *S. scabiei* invade the host and promoting the incidence and development of the host's inflammatory response. This study offers new insights into *S. scabiei* prevention and therapy.

## Data availability statement

The original contributions presented in the study are included in the article/Supplementary material, further inquiries can be directed to the corresponding author.

## Ethics statement

The animal study was approved by the Sichuan Agricultural University Animal Care and Use Committee (SYXK2019-189). The study was conducted in accordance with the local legislation and institutional requirements.

## Author contributions

RH conceived and designed the experiments. RH and QZ performed the experiments and analyzed the data. RH and ZS wrote the manuscript. LX, MG, XG, YX, and JX provided material. ZS improved the paragraphs and English grammar and made constructive suggestions for revisions.
